# Src kinase inhibition promotes the chondrocyte phenotype

**DOI:** 10.1186/ar2308

**Published:** 2007-10-10

**Authors:** Laura Bursell, Anita Woods, Claudine G James, Daphne Pala, Andrew Leask, Frank Beier

**Affiliations:** 1 Department of Physiology and Pharmacology, Canadian Institutes of Health Research Group in Skeletal Development and Remodeling, Schulich School of Medicine and Dentistry, University of Western Ontario, London, Ontario, Canada, N6A 5C1; 2 Department of Oral Biology, Canadian Institutes of Health Research Group in Skeletal Development and Remodeling, Schulich School of Medicine and Dentistry, University of Western Ontario, London, Ontario, Canada, N6A 5C1

## Abstract

Regulated differentiation of chondrocytes is essential for both normal skeletal development and maintenance of articular cartilage. The intracellular pathways that control these events are incompletely understood, and our ability to modulate the chondrocyte phenotype *in vivo *or *in vitro *is therefore limited. Here we examine the role played by one prominent group of intracellular signalling proteins, the Src family kinases, in regulating the chondrocyte phenotype. We show that the Src family kinase Lyn exhibits a dynamic expression pattern in the chondrogenic cell line ATDC5 and in a mixed population of embryonic mouse chondrocytes in high-density monolayer culture. Inhibition of Src kinase activity using the pharmacological compound PP2 (4-Amino-5-(4-chlorophenyl)-7-(t-butyl)pyrazolo [3,4-d]pyrimidine) strongly reduced the number of primary mouse chondrocytes. In parallel, PP2 treatment increased the expression of both early markers (such as Sox9, collagen type II, aggrecan and xylosyltransferases) and late markers (collagen type X, Indian hedgehog and p57) markers of chondrocyte differentiation. Interestingly, PP2 repressed the expression of the Src family members Lyn, Frk and Hck. It also reversed morphological de-differentiation of chondrocytes in monolayer culture and induced rounding of chondrocytes, and reduced stress fibre formation and focal adhesion kinase phosphorylation. We conclude that the Src kinase inhibitor PP2 promotes chondrogenic gene expression and morphology in monolayer culture. Strategies to block Src activity might therefore be useful both in tissue engineering of cartilage and in the maintenance of the chondrocyte phenotype in diseases such as osteoarthritis.

## Introduction

Chondrocytes are the only cell type in cartilage and are predominantly derived from mesenchymal precursor cells. Tight regulation of chondrocyte differentiation is essential both for normal skeletal development and growth, and for the maintenance of joint health (for example, the prevention of degenerative diseases such as osteoarthritis [OA]). The majority of our skeleton develops through the process of endochondral ossification, which starts with the formation of a cartilage template [1-3]. Within this template, chondrocyte proliferation, differentiation (hypertrophy) and apoptosis are precisely regulated, resulting in endochondral bone growth and ultimately replacement of cartilage by bone tissue. Gene mutations and other factors that disturb the normal maturation pattern of chondrocytes generally result in chondrodysplasias and other forms of dwarfism and skeletal deformities [4]. Strict control of the chondrocyte phenotype is also required to maintain the function of the articular cartilage and to prevent cartilage degradation in diseases such as OA. Both loss of the differentiated phenotype and ectopic hypertrophic differentiation are thought to contribute to OA progression [5-7].

Marker genes for chondrocytes of different maturation stages have been identified. Proliferating and articular chondrocytes exhibit fairly similar gene expression patterns; for example, both express Sox9 (a transcription factor that is absolutely necessary for chondrocyte differentiation), the related factors L-Sox5 and Sox6, and collagen type II and aggrecan. Additionally, differentiating chondrocytes produce glycosaminoglycans that are attached to proteoglycans such as aggrecan by xylosyltransferases 1 and 2 (encoded by the *Xylt1 *and *Xylt2 *genes, respectively) [8-10]. Glycosaminoglycans require sulphation for function, a step that is catalyzed by chondroitin sulphotransferases. In cartilage, chondroitin 6 sulphotransferase (encoded by *Chst3*) and chondroitin 4 sulphotransferase (*Chst11*) are of particular importance, as documented by the consequences of mutations in these genes in humans or mice [11,12]. We demonstrated regulation of both xylosyltransferase and chondroitin sulphotransferase genes during chondrogenesis [13,14]. In contrast to these markers of early chondrogenesis, postmitotic and hypertrophic chondrocytes express many different genes, including those encoding collagen type X and the secreted signalling protein Indian hedgehog [4,15,16].

A greater understanding of the mechanisms that control chondrocyte differentiation is required so that we may design efficient strategies to treat skeletal growth disorders, to prevent loss of cartilage in OA and to generate new cartilage in tissue engineering approaches. In particular, loss of the chondrocyte phenotype has been observed both during *in vitro *culture [17-20], complicating tissue engineering approaches to generate cartilage, and during the development of OA *in vivo *[6,21]. However, the signalling pathways that control chondrocyte physiology are only incompletely understood. Tyrosine kinases form one major group of signalling proteins in eukaryotes and can be further divided into two classes [22,23]: the receptor tyrosine kinases and the nonreceptor tyrosine kinases. The former are cell surface receptors for extracellular ligands such as growth factors. Several of them, most notably fibroblast growth factor receptor 3 [24], have been shown to play important roles in chondrocytes. In contrast, very little is known about the function of nonreceptor tyrosine kinases in cartilage. This study focuses on one prominent family within this class, the Src kinases.

The Src family consists of 11 members that have been implicated in many cellular functions, including cell proliferation, differentiation, apoptosis and migration [25,26]. Most mammalian cells express multiple family members with overlapping functions. The resulting redundancy has created problems in investigating the function of individual Src kinase family members, because genetic inactivation (for example, in knockout mice) of individual members has often yielded surprisingly mild phenotypes. To overcome this problem, Src family function is often studied using PP2 (4-Amino-5-(4-chlorophenyl)-7-(t-butyl)pyrazolo [3,4-d]pyrimidine), a pharmacological compound that inhibits all Src kinases [27,28]. In this study, we employed PP2 to examine the effects of Src inhibition on chondrocyte differentiation. We specifically considered whether and how PP2 would affect parameters of the chondrocyte phenotype, such as chondrogenic morphology, actin organization and gene expression.

## Materials and methods

### Cell culture

ATDC5 cells were cultured in media consisting of 47.5% Dulbecco's modified essential medium, 47.5% F12, 5% foetal bovine serum, 0.25% L-glutamine and 0.25% Penstrep (Invitrogen, Burlington, Ontario, Canada), and induced to differentiate with 1% ITS (Sigma, Oakville, Ontario, Canada), as described previously [29,30]. Cells for RNA isolation were plated in six-well plates (Falcon) at a density of 60,000 cells/well.

For primary chondrocyte cultures, embryos were dissected from CD1 timed-pregnant mice (Charles River Laboratories, St. Constant, Quebec, Canada) at embryological day 15.5, and chondrocytes were isolated from long bones, as described previously [31,32], by sequential digestion with trypsin and collagenase. Cells were re-suspended in media consisting of 55% F12, 35% Dulbecco's modified essential medium and 10% foetal bovine serum, and supplemented with 0.25% L-glutamine and 0.25% Penstrep (Invitrogen). The primary chondrocytes were then plated according to the desired use. For RNA and protein isolation, chondrocytes were plated in six-well plates (Falcon) at 500,000 cells per well. Primary chondrocyte cultures were treated with 1 μmol/l or 10 μmol/l of the Src family kinase inhibitor PP2 (Calbiochem; VWR, Mississauga, Ontario, Canada) or with dimethyl sulphoxide (DMSO; Sigma), the vehicle in which PP2 was dissolved, as control. For other experiments, primary chondrocytes were cultured as described above and treated with 10 μmol/l Y27632, 1 μmol/l cytochalasin D, 50 nmol/l jasplakinolide, or the vehicle DMSO.

### Taqman real-time PCR

Total RNA was harvested from treated cultures on days 3, 6, 9, 12, 15, and 18 of ATDC5 differentiation, as well as from primary chondrocyte cultures treated with DMSO, PP2, cytochalasin D, Y27632, or jasplakinolide. RNA was isolated using a Qiagen (Mississauga, Ontario, Canada) RNeasy kit, following the manufacturer's instructions. RNA samples were diluted to a final concentration of 25 ng/μl for use in real-time RT-PCR, as described previously [33]. Relative gene expression was measured using Assays on Demand (Applied Biosystems) for *Col2a1*, *Col10a1*, *Agc1*, *Sox6*, *Sox5*, *Sox9*, *Chst3*, *Chst11*, *Xylt1*, *Xylt2*, *Ihh*, *Cdkn1c*, *Atf3*, *Lyn*, *Frk*, and *Hck *in relation to the glyceraldehyde-3-phosphate dehydrogenase gene (*Gapdh*; Applied Biosystems, Foster City, CA, USA) using One Step RT qPCR Master Mix Plus (Eurogentec North America, San Diego, CA, USA) and 40 cycles on the ABI Prism 7900 HT sequence detector (PerkinElmer, Emeryville, CA, USA). Real-time analysis was performed as quadruplicate reactions on at least three independent trials in each experimental situation.

### Western blotting

Protein was harvested from primary cell cultures using RIPA lysis buffer (150 nmol/l NaCl, 50 mmol/l Tris-HCl [pH 7.5], 1% Triton-X, 1% deoxycholate, 0.1% SDS, 2 mmol/l EDTA) supplemented with a protease inhibitor mini complete tablet (Roche Applied Science, Laval, Quebec, Canada). The isolated protein was then sonicated and quantified with the Bicinchoninic Acid kit (Sigma), in accordance with the manufacturer's protocol. An equal quantity (20 μg) of each sample was used in SDS-PAGE and transferred to nitrocellulose membrane (BioRad Labratories, Mississauga, Ontario, Canada). Membranes were then blocked in 5% bovine serum albumin (BSA) in Tris-buffered saline with 0.01% Tween-20 (TBST), followed by overnight incubation at 4°C with primary antibody against Lyn (Cell Signaling Technology, Inc., Mississauga, Ontario, Canada) or β-actin (Sigma) diluted 1:200 in 5% bovine serum albumin-TBST. Blots were then washed three times for 5 minutes in TBST, followed by incubation with the appropriate HRP (horseradish peroxidase)-conjugated secondary antibody (Santa Cruz Biotechnology, Santa Cruz, CA, USA) for 1 hour at 4°C. Membranes were then washed three times for 5 minutes in TBST before detection of proteins by ECL Western Blot detection reagents (Amersham Bioscience, Oakville, Ontario, Canada), in accordance with the manufacturer's instructions. Banding on the membranes was visualized using a ChemiImager 5500 (AlphaInnotech Inc., San Leandro, CA, USA). Western blotting was performed on samples from three independent trials.

### MTT assays

Cellular proliferation was determined using an MTT Cell Proliferation Kit I (Roche Applied Science), which had been tested and confirmed as a valid method for quantifying chondrocyte cell number in our laboratory [29,34]. Primary chondrocytes were plated on 96-well plates at a density of 10,000 cells/well and treated with 1 μmol/l or 10 μmol/l of the tyrosine kinase inhibitor Tyr A23, Tyr A25, or Tyr A47, or the Src family kinase inhibitor PP2, or with DMSO alone as the vehicle control. In accordance with the manufacturer's directions, cells were treated with MTT (3-[4,5-dimethylthiazol-2-yl]-2,5-diphenyl tetrazolium bromide) at 24, 48 and 72 hours and, following incubation and solubilization, absorbance was measured at 600 nm with a spectrophotometer as described previously [35]. MTT assays were performed in four independent trials.

### Immunofluorescence

Primary chondrocytes were plated on glass coverslips in a 24-well plate at a density of 50,000 cells/well and cells were allowed to adhere overnight. The following morning, cells were treated either with 10 μmol/l PP2 or DMSO as a control and incubated for an additional 24 hours. The next day, cells were freshly treated with the inhibitor or vehicle alone. After a 1-hour incubation period, cells were washed with room temperature phosphate-buffered saline (PBS) and fixed with 4% paraformaldehyde in PBS for 10 minutes. After rinsing with fresh PBS, cells were incubated with 0.1% Triton-X solution in PBS for 5 minutes to allow for membrane permeability. The coverslips were then rinsed in PBS and incubated in blocking solution (1:20 goat serum [Sigma]:PBS) for 30 minutes at room temperature. After blocking, primary antibody against FAK (BioSource International, Inc.) or phospho-FAK [pY397] (BioSource International, Inc., Montreal, Quebec, Canada; 1:100 antibody:blocking solution) was added to the coverslips and incubated for 45 minutes at room temperature. After washing with PBS, the coverslips were incubated for 45 minutes in the dark at room temperature with a fluorescein isothionate-conjugated secondary antibody diluted 1,000 times in PBS. Coverslips were again washed before incubation with rhodamine-phalloidin solution (Cytoskeleton, Denver, CO, USA) for 45 minutes in a dark environment. Following incubation, the coverslips were rinsed and mounted using VectaShield with DAPI (Vector Laboratories Inc., Burlingane, CA, USA). Images of these cells were taken with a Leica DMRA2 fluorescence microscope (Quorum Technologies, Guelph, Ontario, Canada) at 63-fold magnification and analyzed using OpenLab software. (Quorum Technologies, Guelph, Ontario, Canada). Fluorescence imaging was performed on at least three independent trials.

### RhoA activity assay

Primary chondrocytes were cultured with DMSO or PP2 for 1 to 3 days. Ras homology A (RhoA) activity was measured using a luminescent G-LISA™ kit (Cytoskeleton), as described previously [36].

### Statistical analyses

All experiments were performed in at least three independent trials. Real-time PCR data represent an average of three independent trials of samples run in quadruplicate, normalized to *Gapdh *and to day control (DMSO) levels. Statistical significance was determined using a one-way analysis of variance with Bonferroni's post-test (*P *< 0.05). Statistical tests were performed using GraphPad Prism version 4.00 for Windows (GraphPad Software, San Diego, CA, USA).

## Results

### Dynamic expression of *Lyn *during chondrocyte differentiation

**Figure 1:**
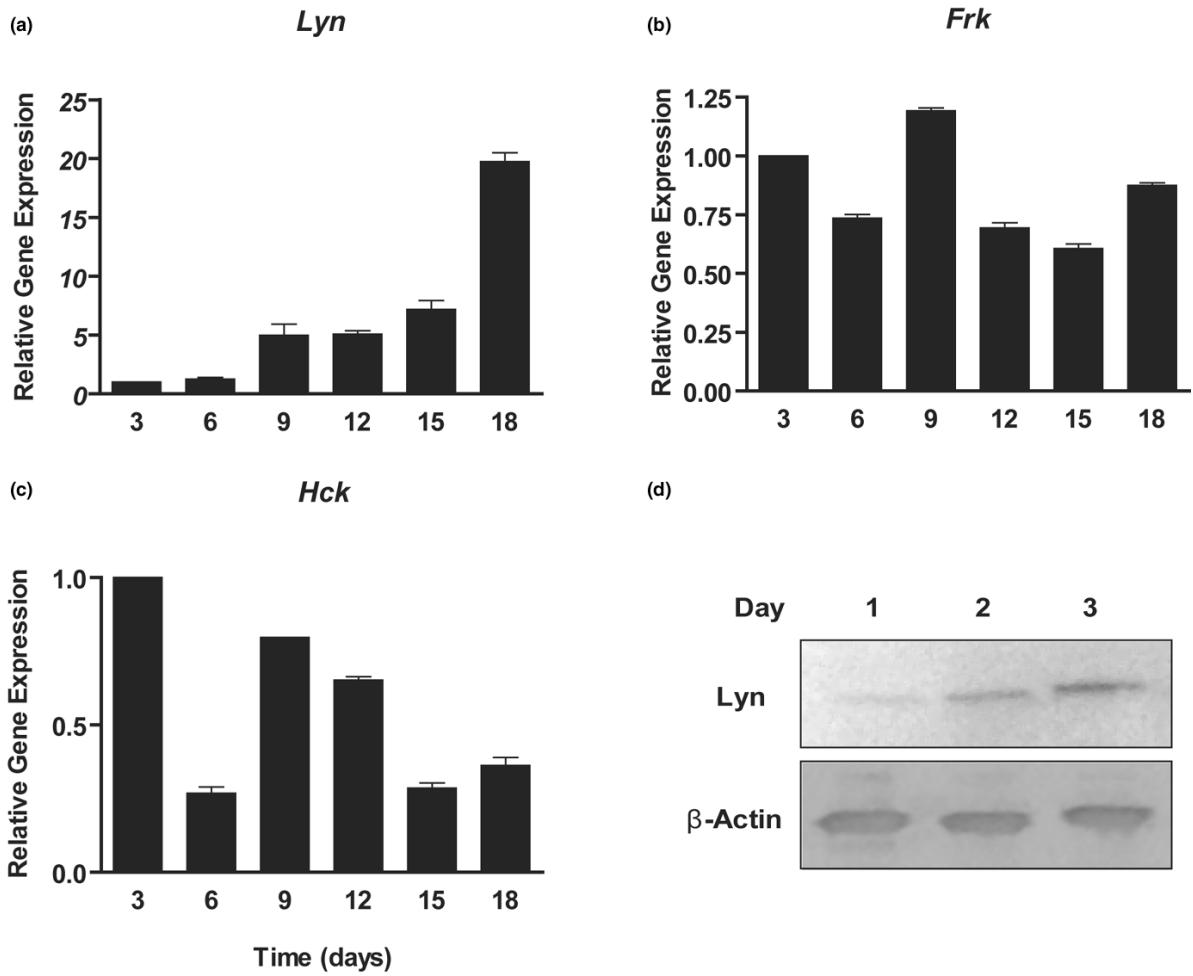
Expression of Src kinases in chondrogenic cells. Expression of **(a) ***Lyn*, **(b) ***Frk *and **(c) ***Hck *Src kinase genes during chondrogenic differentiation of ATDC5 cells (days 3 to 18) was analyzed using Taqman real-time PCR analyses. *Lyn *mRNA levels increased strongly during differentiation, whereas the other two kinase genes exhibited more subtle changes in gene expression. **(d) **Western blotting confirmed expression of Lyn protein in primary mouse chondrocytes in monolayer culture for 1 to 3 days.

We first examined the expression of Src family members in a microarray dataset derived from embryonic limb bud mesenchymal cells undergoing chondrogenic differentiation in micromass culture [37]. The *Lyn*, *Hck *and *Frk *genes exhibited significant expression during chondrocyte differentiation and were chosen for further study. The ATDC5 cell line has been shown to undergo the entire process of chondrocyte differentiation when treated with insulin [38,39]. Real-time PCR demonstrated that *Lyn *expression increased strongly throughout ATDC5 differentiation (Figure 1a). In contrast, *Frk *and *Hck *transcript levels underwent relatively minor changes during ATDC5 differentiation (Figure 1b,c).

High-density monolayer cultures of primary chondrocytes isolated from embryonic growth plates present a mixed population expressing both early and late markers of chondrocyte differentiation. Primary chondrocyte cultures are a good model in which to study chondrocyte physiology because they are closer to the *in vivo *chondrocyte phenotype than are transformed cell lines. Western blotting confirmed that Lyn protein is expressed in primary mouse chondrocytes in monolayer culture and increases over the culture period (Figure 1d).

### Src inhibition decreases chondrocyte cell numbers

**Figure 2:**
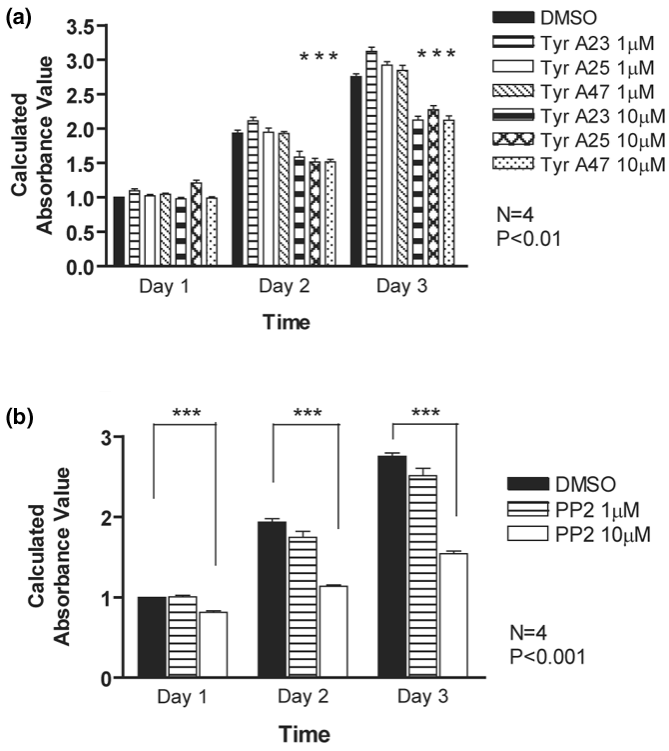
Src kinase activity regulates chondrocyte cell numbers. Primary mouse chondrocytes were incubated for 1 to 3 days with dimethyl sulphoxide (DMSO), **(a) **the general tyrosine kinase inhibitors Tyr A23, Tyr A25 and Tyr A47, or **(b) **the Src inhibitor PP2 (1 and 10 μmol/l each). Cell numbers were determined by MTT assay. All inhibitors reduced cell numbers at the 10 μmol/l concentration, but the effects of PP2 were more pronounced (*n *= 4; *P < 0.01, ***P < 0.001).

We next examined effects of general tyrosine kinase and Src-specific inhibitors on numbers of primary mouse chondrocytes using MTT assays, because our earlier studies had demonstrated that MTT values correspond to chondrocyte cell numbers [29,34]. Over the 3-day time course, numbers of chondrocytes in control cultures increased steadily. The general tyrosine kinase inhibitors Tyr A23, Tyr A25 and Tyr A47 had minor effects on cell number at 1 μmol/l concentration, but they caused a significant decrease in cell numbers at 10 μmol/l (Figure 2a). In comparison, the more specific compound PP2 that only inhibits the Src family of tyrosine kinases caused a much stronger reduction in cell number, in particular at the 10 μmol/l concentration (Figure 2b). These data suggest that Src kinases are required for proliferation of these cells.

### Src inhibition promotes chondrogenic gene expression

**Figure 3:**
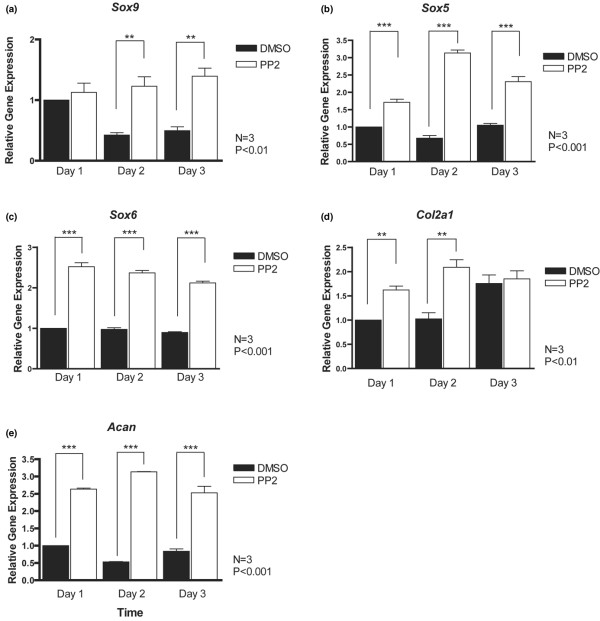
PP2 promotes expression of early chondrocyte marker genes. Primary mouse chondrocytes were incubated for 1 to 3 days with dimethyl sulphoxide (DMSO) or the Src inhibitor PP2 (10 μmol/l), and transcript levels of early chondrocyte marker genes were determined by real-time PCR. Expression levels of **(a) ***Sox9*, **(b) ***Sox5*, **(c) ***Sox6*, **(d) ***Col2a1 *and **(e) ***Acan *(aggrecan) were significantly increased upon Src inhibition (*n *= 3; **P < 0.01, ***P < 0.001).

We then examined the effects of PP2 (10 μmol/l) on the expression of chondrocyte marker genes using the same culture system. In control cultures, levels of transcripts for the chondrogenic master transcription factor Sox9 decreased slightly over the culture period (Figure 3a). This downregulation was prevented by PP2. mRNA levels for the related transcription factors L-Sox5 and Sox6 remained constant over 3 days in control cultures but were significantly enhanced by PP2 (Figure 3b,c). Similar trends were observed for the major extracellular matrix genes *Col2a1 *(which encodes collagen type II) and *Acan *(which encodes aggrecan; Figure 3d,e), but *Acan *induction by PP2 was markedly greater than that of the other genes.

**Figure 4:**
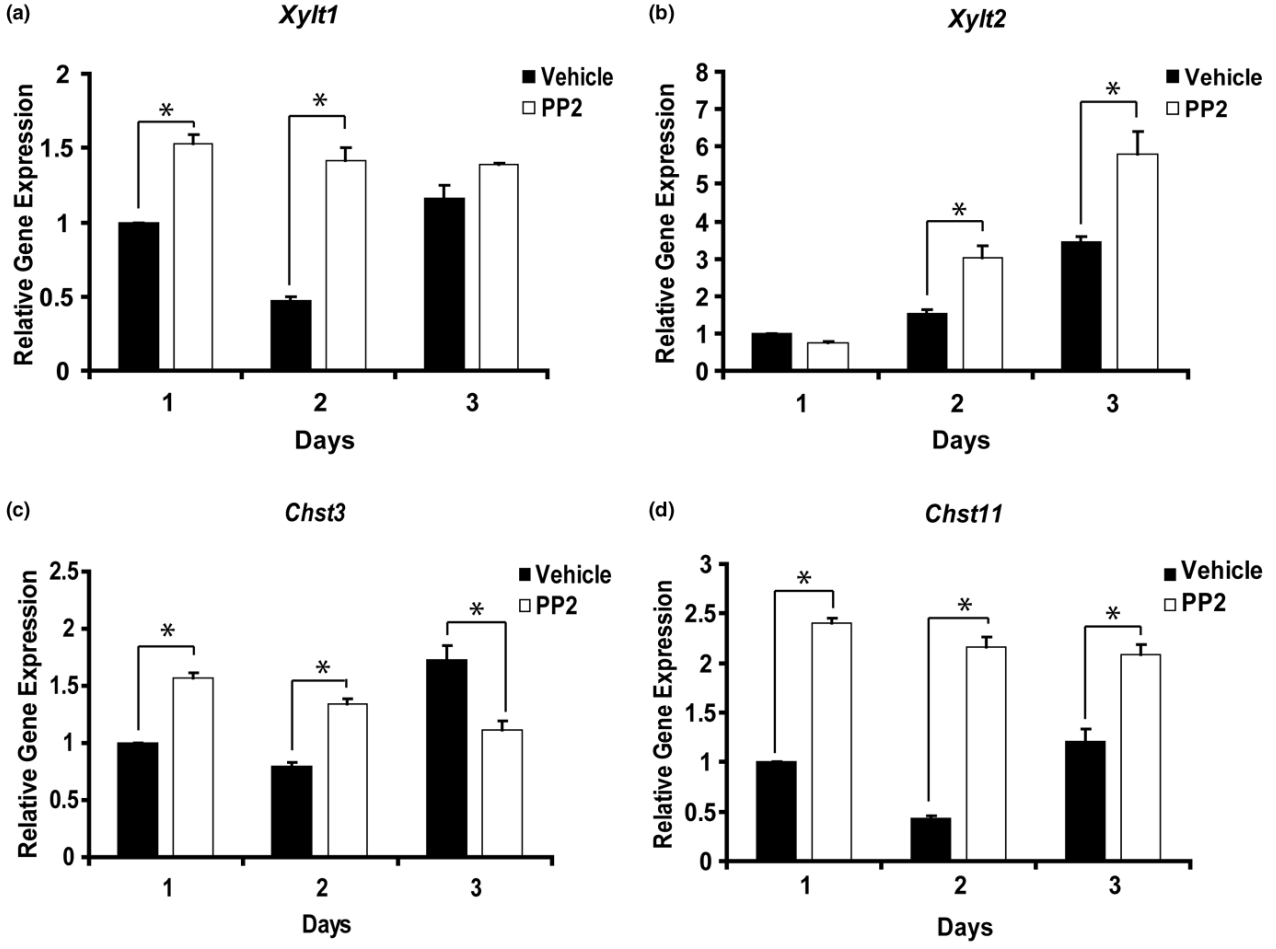
PP2 promotes expression of genes involved in glycosaminoglycan synthesis. Primary mouse chondrocytes were incubated for 1 to 3 days with dimethyl sulphoxide or the Src inhibitor PP2 (10 μmol/l), and transcript levels of genes involved in glycosaminoglycan synthesis were determined by real-time PCR. Expression levels of **(a) ***Xylt1*, **(b) ***Xylt2*, **(c) ***Chst3 *and **(d) ***Chst11 *were significantly increased upon Src inhibition (*n *= 3; *P < 0.05).

Because aggrecan is the major core proteoglycan in cartilage, we wished to determine whether PP2 affects genes that are involved in glycosaminoglycan synthesis. Transcripts for the xylosyltransferases were significantly increased at days 1 and 2 (*Xylt1*) or days 2 and 3(*Xylt2*) in response to Src family inhibition (Figure 4a,b). Additionally, both examined chondroitin sulphotransferase genes (*Chst3 *and *Chst11*) were significantly upregulated in primary chondrocyte cultures by PP2 treatment for 1 and 2 days (Figure 4c,d). However, *Chst3 *expression decreased by day 3 in cultures treated with PP2 in comparison with control, whereas *Chst11 *levels remain elevated in the presence of PP2 at day 3.

**Figure 5:**
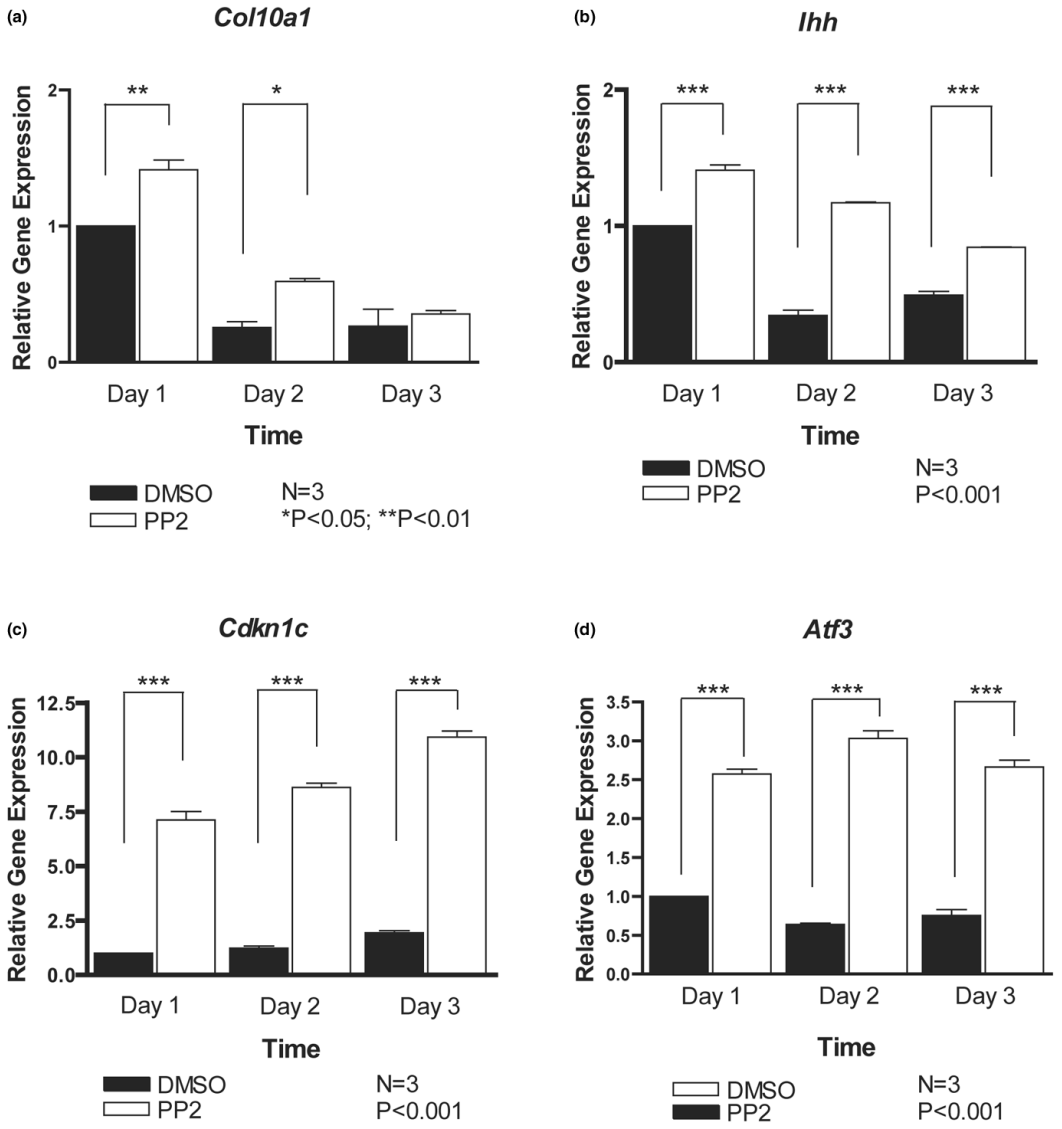
PP2 promotes expression of late chondrocyte marker genes. Primary mouse chondrocytes were incubated for 1 to 3 days with dimethyl sulphoxide (DMSO) or the Src inhibitor PP2 (10 μmol/l), and transcript levels of late chondrocyte marker genes were determined by real-time PCR. Expression levels of **(a) ***Col10a1*, **(b) ***Ihh*, **(c) ***Cdkn1c *and **(d) ***Atf3 *were significantly increased upon Src inhibition (*n *= 3; *P < 0.05, **P < 0.01, ***P < 0.001).

We also assessed effects of Src inhibition on markers for later (postmitotic) stages of chondrocyte maturation. Both *Col10a1 *(collagen type X) and *Ihh *(Indian hedgehog) mRNA levels decreased over time in culture, but were higher upon PP2 treatment (Figure 5a,b). Similarly, PP2 increased the expression of transcripts for the cell cycle inhibitor p57 (*Cdkn1c*) and for *Atf3 *(Figure 5c,d), which encodes a transcription factor that we have identified as being upregulated during chondrocyte hypertrophy [31]. Thus, the expression levels for all 13 chondrocyte marker genes examined here were elevated in the presence of PP2.

### PP2 causes reduced expression of *Lyn*, *Frk *and *Hck*

**Figure 6:**
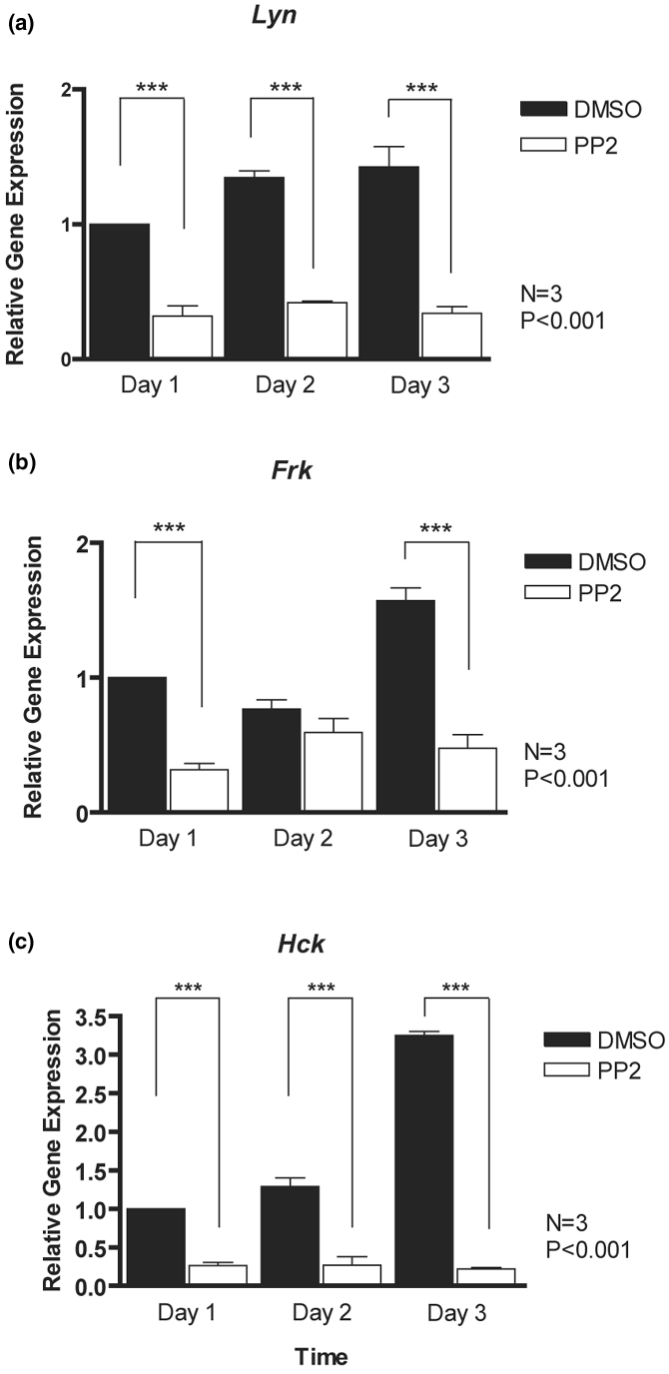
PP2 represses expression of Src kinase genes. Primary mouse chondrocytes were incubated for 1 to 3 days with dimethyl sulphoxide (DMSO) or the Src inhibitor PP2 (10 μmol/l), and transcript levels of Src kinase genes were determined by real-time PCR. Expression levels of **(a) ***Lyn*, **(b) ***Frk *and **(c) ***Hck *were significantly decreased upon Src inhibition (*n *= 3; ***P < 0.001).

We considered whether PP2 also influences expression of the Src kinase genes in chondrocytes. All three examined Src kinase genes (*Lyn*, *Frk *and *Hck*) exhibited upregulation over the 3-day culture period in control conditions (Figure 6a–c). However, transcript levels were significantly reduced in the presence of PP2 at all stages (with the exception of *Frk *at day 2). These data suggest that Src activity is required to maintain expression of at least some family members.

### Src inhibition results in cell rounding and a reduction in stress fibres

**Figure 7:**
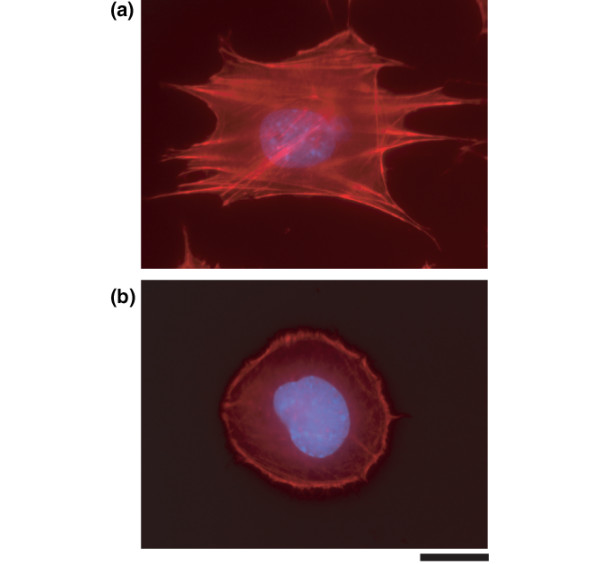
PP2 promotes cell rounding and cortical actin formation. Primary mouse chondrocytes were incubated for 24 hours with **(a) **dimethyl sulphoxide or **(b) **the Src inhibitor PP2 (10 μmol/l), and cells were stained with rhodamine-phalloidin (red) for polymerized actin and with DAPI for nuclei (blue). PP2 induced cell rounding, loss of stress fibre, and cortical organization of actin (scale bar: 2 μm).

**Figure 8:**
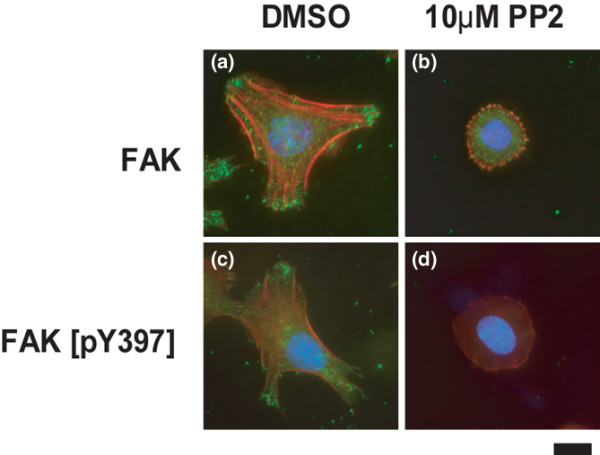
PP2 promotes cell rounding and cortical actin formation. Primary mouse chondrocytes were incubated for 24 hours with dimethyl sulphoxide (DMSO) or the Src inhibitor PP2 (10 μmol/l), and cells were stained with antibodies against total focal adhesion kinase (FAK) or FAK phosphorylated on residue tyrosine 397 (green), rhodamine-phalloidin (red) and DAPI (blue). In the presence of DMSO, total and phosphorylated actin localized to focal adhesions at the end of stress fibres. In cells treated with PP2, total FAK acquired a diffuse cytosolic staining, whereas the signal for phosphorylated FAK was greatly reduced (scale bar: 2 μm).

During our studies, we noticed that PP2 induces a marked change in chondrocyte morphology. In monolayer culture, chondrocytes rapidly lose their rounded/polygonal shape, become elongated and fibroblastoid, and develop prominent actin stress fibres (Figure 7a[20]). PP2 treatment reversed these effects and resulted in rounded cells with a cortical actin cytoskeleton, without stress fibres (Figure 7b). Stress fibre formation correlated with the accumulation of focal adhesion kinase (FAK) at the ends of the fibres (Figure 8a). Immunofluorescence for a phosphorylated form of FAK (Y397) exhibited a similar pattern (Figure 8c). In the presence of PP2, total FAK showed diffuse staining (Figure 8b) and phosphorylated FAK was no longer detectable (Figure 8d), suggesting that PP2 inhibits FAK phosphorylation.

**Figure 9:**
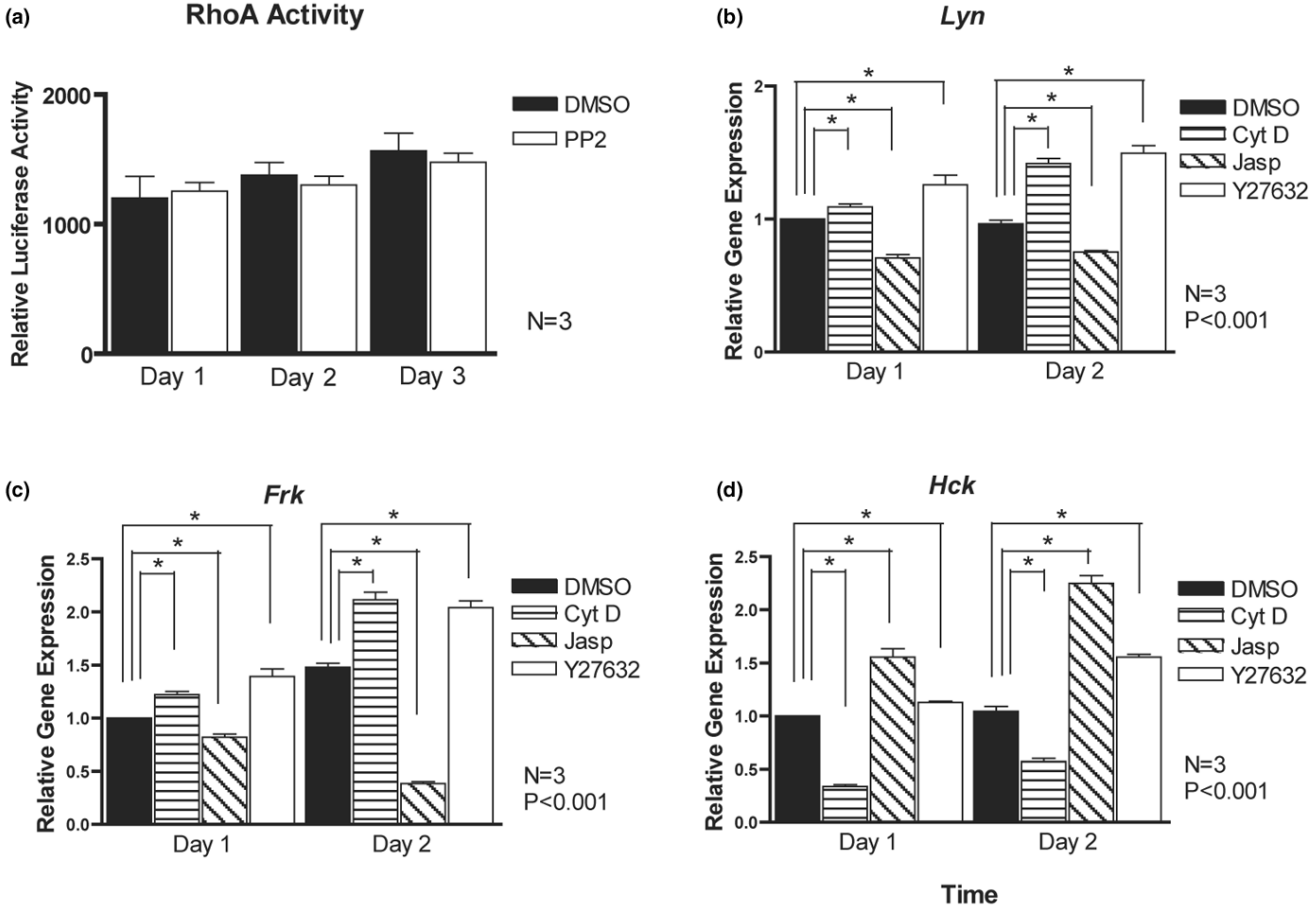
Interplay between RhoA/ROCK and Src kinase signalling. **(a) **Primary mouse chondrocytes were incubated for 1 to 3 days with dimethyl sulphoxide (DMSO) or the Src inhibitor PP2 (10 μmol/l), and Ras homology A (RhoA) activity was measured using the G-LISA™ kit (Cytoskeleton). No significant differences in RhoA activity were observed upon Src inhibition (*n *= 3). **(b) **Primary mouse chondrocytes were incubated for 1 or 2 days with DMSO or the Rho-associated, coiled-coil containing protein kinase (ROCK) inhibitor Y27632 (10 μmol/l) or the actin-modifying drugs cytochalasin D (1 μmol/l) and jasplakinolide (50 nmol/l). Expression levels of **(b) ***Lyn*, **(c) ***Frk *and **(d) ***Hck *genes were determined using real-time PCR, which demonstrated regulation of all three genes by the employed drugs (*n *= 3, *P < 0.001).

The morphological changes that occurred upon PP2 treatment resembled those we had observed when we inhibited Rho-associated, coiled-coil containing protein kinase (ROCK)1/2 kinases, prime effectors of the small GTPase RhoA [20]. These data suggest that Src kinases and RhoA/ROCK might act in the same signalling pathway. We therefore considered whether PP2 treatment modulates RhoA activity. Primary chondrocytes were treated with PP2 for 1 to 3 days, and RhoA activity was determined as described previously [36]. Src inhibition did not affect RhoA activity at any stage (Figure 9a). To determine whether ROCK signalling affects expression of the Src kinases studied here, we analyzed *Lyn, Frk *and *Hck *transcript levels in primary chondrocytes treated with the ROCK inhibitor Y27632. Parallel experiments were done with the actin-modifying drugs cytochalasin D and jasplakinolide, which have overlapping effects with Y27632 on chondrocytes [20,36]. Both *Lyn *and *Frk *transcripts were slightly increased in response to Y27632 and cytochalasin D and repressed by jasplakinolide (Figure 9b,c). In contrast, *Hck *transcript levels were reduced by cytochalasin D and enhanced by jasplakinolide and Y27632 (Figure 9d).

## Discussion

In this study we demonstrate that the Src inhibitor PP2 reduces proliferation, promotes chondrogenic gene expression and induces chondrogenic morphology in primary chondrocytes in monolayer culture. These data suggest that inhibition of Src activity could be a valid strategy to maintain or induce the chondrocyte phenotype, for example in tissue engineering approaches to *in vitro *cartilage formation or in the prevention of cartilage loss in OA patients. Moreover, our data suggest physiological roles for Src kinases in the regulation of chondrogenesis.

General inhibition of tyrosine kinase activity by a variety of compounds resulted in a mild reduction in chondrocyte cell numbers. Our data cannot distinguish whether these effects are due to decreased proliferation or increased cell death; however, because these effects were observed while cell numbers increased rapidly, a proliferative effect appears more likely. Specific inhibition of one subgroup of tyrosine kinases, the Src family, caused a much more marked reduction in cell numbers. These data suggest that other tyrosine kinases counteract the mitogenic effects of Src proteins. One likely candidate for such a function is the receptor tyrosine kinase fibroblast growth factor receptor 3, which is known to induce cell cycle withdrawal in chondrocytes [24]. However, it is likely that other promitogenic and antimitogenic tyrosine kinases also contribute to the effects of the broad spectrum tyrosine kinase inhibitors used here. For example, mouse knockout studies suggest that the receptor tyrosine kinases discoidin domain receptor 2 and receptor tyrosine kinase-like orphan receptor 2 regulate normal chondrocyte proliferation [40,41].

We have demonstrated expression of three Src kinases – Lyn, Frk and Hck – in chondrocytes. It is likely that additional family members are present as well. These data suggest that the effects of PP2 observed in our experiments are due to inhibition of multiple Src kinases. Because of overlapping expression patterns and the well established functional redundancy within the Src family, identification of the roles played by individual family members in chondrocytes will be a major challenge and will probably require detailed analyses of knockout mice for single or multiple Src family genes.

Several of the effects of PP2 observed here were strikingly similar to those obtained upon inhibition of the RhoA/ROCK pathway that we described previously [20,36,42]. For example, inhibition of ROCK kinase activity resulted in cell rounding, loss of stress fibres and increased expression of Sox9, collagen type II and aggrecan in chondrocyte monolayer cultures. Similarly, inhibition of ROCK activity increased the expression of hypertrophic markers such as collagen type X in differentiating chondrocytes [29]. These data suggest that RhoA/ROCK and Src kinases could be part of the same pathway that regulates chondrocyte differentiation. Our data show that PP2 does not affect RhoA activity in chondrocytes, suggesting that Src kinases do not act upstream of RhoA. ROCK inhibition caused a slight increase in the expression of *Lyn *and *Frk *transcripts, suggesting that RhoA/ROCK does not act primarily by suppressing the expression of these Src kinase genes, at least at the RNA level. However, these data do not exclude the possibility that the Rho pathway controls activity of Src kinases at the post-transcriptional level. In addition, it is feasible that both signalling systems act in parallel pathways. Experiments are under way to examine these possibilities.

Another protein that probably interacts with Src kinases in the control of chondrocyte physiology is FAK. Our data show that reduced FAK phosphorylation is associated with the loss of stress fibres and cell rounding in response to PP2. FAK is a direct substrate of Src kinases, but the residue examined (Y397) is auto-phosphorylated by FAK in response to integrin engagement [43,44]. Therefore, reduced Y397 phosphorylation is not a direct effect of Src inhibition, but is secondary to other events. Nevertheless, FAK might constitute a direct link between Src activity and chondrocyte morphology, but this remains to be proved. It will also be of interest to examine whether FAK is involved in the gene expression changes induced by PP2, because chondrocyte cell shape and gene expression are closely linked [42].

Our results also demonstrate that several genes involved in glycosaminoglycan synthesis (*Xylt1*, *Xylt2*, *Chst3 *and *Chst11*) are regulated by PP2 in a manner similar to classical chondrocyte markers such as collagen type II and aggrecan. We observed similar co-regulation in our recent studies on control of chondrogenesis by Rac1 [13] and C-type natriuretic peptide [14]. These data suggest that these genes (and potentially others that are involved in glycosaminoglycan synthesis) should be included in the list of parameters investigated during studies of chondrogenesis and might present novel markers of chondrocyte differentiation.

One of the surprises of this study was the repression of *Lyn*, *Frk *and *Hck *transcript levels by PP2 treatment. It therefore appears that PP2 not only reduces the enzymatic activity of Src kinases directly but also inhibits expression of selected family members. It remains to be seen whether similar mechanisms exist in other tissues or for additional Src kinases.

## Conclusion

In summary, we have demonstrated that Src inhibition promotes the chondrocyte phenotype by inducing chondrogenic cell shape and gene expression. These data both demonstrate physiological roles of Src family kinases in cartilage development and homeostasis, and suggest their potential as therapeutic targets in the treatment of skeletal diseases and in the generation of cartilage in tissue-engineering approaches.

## Abbreviations

DMSO = dimethyl sulphoxide; FAK = focal adhesion kinase; MTT = 3-[4,5-dimethylthiazol-2-yl]-2,5-diphenyl tetrazolium bromide; OA = osteoarthritis; PBS = phosphate-buffered saline; PP2 = 4-Amino-5-(4-chlorophenyl)-7-(t-butyl)pyrazolo [3,4-d]pyrimidine; RhoA = Ras homology A; ROCK = Rho-associated, coiled-coil containing protein kinase; RT-PCR = reverse transcription polymerase chain reaction; TBST = Tris buffered saline with 0.01% Tween-20.

## Competing interests

The authors declare that they have no competing interests.

## Authors' contributions

LB performed most experiments and their analyses. AW, CJG, DP and AL contributed data, samples and expertise. FB conceived of and designed the study and co-wrote the manuscript with LB and AW. All authors read and approved the manuscript.
